# Factors affecting accuracy of estimated effective number of chromosome segments for numerically small breeds

**DOI:** 10.1111/jbg.12512

**Published:** 2020-10-10

**Authors:** Jovana Marjanovic, Mario P. L. Calus

**Affiliations:** ^1^ Animal Breeding and Genomics Wageningen University & Research Wageningen The Netherlands

**Keywords:** cattle, genomic prediction, independent chromosome segments, multi‐breed, small breed

## Abstract

For numerically small breeds, obtaining a sufficiently large breed‐specific reference population for genomic prediction is challenging or simply not possible, but may be overcome by adding individuals from another breed. To prioritize among available breeds, the effective number of chromosome segments (*M*_e_) can be used as an indicator of relatedness between individuals from different breeds. The *M*_e_ is also an important parameter in determining the accuracy of genomic prediction. The *M*_e_ can be estimated both within a population and between two populations or breeds, as the reciprocal of the variance of genomic relationships. However, the threshold for number of individuals needed to accurately estimate within or between populations *M*_e_ is currently unknown. It is also unknown if a discrepancy in number of genotyped individuals in two breeds affects the estimates of *M*_e_ between populations. In this study, we conducted a simulation that mimics current domestic cattle populations in order to investigate how estimated *M*_e_ is affected by number of genotyped individuals, single‐nucleotide polymorphism (SNP) density and pedigree availability. Our results show that a small sample of 10 genotyped individuals may result in substantial over or underestimation of *M*_e_. While estimates of within population *M*_e_ were hardly affected by SNP density, between population *M*_e_ values were highly dependent on the number of available SNPs, with higher SNP densities being able to detect more independent chromosome segments. When subtracting pedigree from genomic relationships before computing *M*_e_, estimates of within population *M*_e_ were three to four times higher than estimates with genotypes only; however, between *M*_e_ estimates remained the same. For accurate estimation of within and between population *M*_e_, at least 50 individuals should be genotyped per population. Estimates of within *M*_e_ were highly affected by whether pedigree was used or not. For within *M*_e_, even the smallest SNP density (~11k) resulted in accurate representation of family relationships in the population; however, for between *M*_e_, many more markers are needed to capture all independent segments.

## 1 INTRODUCTION

Numerically small breeds often have difficulties to compete with larger and highly performing mainstream breeds, which endangers their existence (Addo et al., 2017; Hiemstra et al., 2010). These small breeds, however, are well worth preserving as they possess unique genetic diversity and show high adaptation to local environments. In other words, they can fulfil a sustainable role in the society (Oldenbroek, 2007). To improve the long‐term perspectives of small breeds, it is necessary to maintain their economic competitiveness and preferably enhance it. In recent years, genomic prediction of breeding values, that is prediction based on marker data alone, revolutionized the field of animal breeding (Meuwissen et al., 2001). In dairy cattle breeding genomic selection significantly reduced the generation interval through selection of animals earlier in their life, which resulted in higher genetic gains per year (Bouquet & Juga, 2013; Pryce et al., 2011). Genomic selection, therefore, can be used in small breeds to improve their competitiveness and economic perspectives for farmers to use these breeds on their farms. In addition, methods such as genomic optimal contribution selection (Sonesson et al., 2012) can be applied to simultaneously assure genetic improvement of the breed and the maintenance of its diversity.

The principle of genomic prediction is that the reference population, which consists of individuals that are both phenotyped and genotyped for thousands of single‐nucleotide polymorphisms (SNPs), is used to estimate SNP effects. The estimated SNP effects are subsequently used to infer genomic estimated breeding values (EBVs) of selection candidates, who only have genotypes. Size of the reference population is one of the key parameters that affects accuracy of genomic prediction (Daetwyler et al., 2008; Meuwissen et al., 2001; VanRaden et al., 2009). For numerically small breeds, however, obtaining a sufficiently large breed‐specific reference population for genomic prediction may be challenging or simply not possible, either because of limited resources available for genetic improvement of the breed, or simply because limited numbers of animals are available within the breed. Adding individuals from other breeds to the reference populations may help to overcome this issue. The benefit of reference individuals from another breed strongly relies on relatedness between the breeds, where higher increase in accuracy is expected when closely related breeds are combined in the reference population, while no or only low increases in accuracy are expected when those breeds are more distant (Brøndum et al., 2011; Habier et al., 2007, 2010; Hozé et al., 2014). To prioritize among available breeds, the effective number of chromosome segments (*M*_e_) can be used as an indicator of relatedness between individuals from different breeds (Wientjes et al., 2016).

The *M*_e_ is an important parameter in determining the accuracy of genomic prediction in breeds with a single‐breed (Goddard, 2009) or multi‐breed reference population (Wientjes et al., 2016). The *M*_e_ can be estimated both within a population and between two populations or breeds. The *M*_e_ within a population describes the number of chromosome segments that are segregating independently in the population. Effects for each of these segments need to be estimated in order to predict genomic breeding values of individuals from a given population (Meuwissen et al., 2013; Wientjes et al., 2016). The accuracy of genomic prediction increases as the number of segment decreases (Daetwyler et al., 2008). The *M*_e_ within a population is directly related to the effective population size (*N*_e_) (Brard & Ricard, 2015; Goddard, 2009; Lee et al., 2017). Low *N*_e_ is associated with higher relatedness among individuals, higher extent of linkage disequilibrium (LD) (Falconer & Mackay, 1996; Sved, 1971) and lower number of segregating chromosome segments. Hence, populations or breeds with similar selection history and LD structure are expected to have similar values of *M*_e_. The *M*_e_ between populations gives insight in the consistency of LD between the two populations (Wientjes et al., 2016). Low *M*_e_ between populations indicates high relatedness between two populations, while between populations that were split more generations ago usually a higher value of *M*_e_ is observed (see general discussion in Wientjes, 2016).

In general, before all genotypes are available for both reference animals and selection candidates, a population parameter such as *M*_e_ can be used to predict the anticipated accuracy of genomic selection (Goddard et al., 2011; Vandenplas et al., 2017; VanRaden, 2008; Wientjes, et al., 2015). The predicted accuracies can then help to decide whether implementation of genomic selection is expected to be beneficial. To keep initial costs minimal, the number of animals to genotype to be able to estimate *M*_e_, and predict the accuracies of genomic selection, should preferably be as small as possible. Previous studies aiming to estimate within and between population *M*_e_ used 100 or more individuals (van den Berg et al., 2015; Erbe et al., 2013; Wientjes, et al., 2015). The threshold for number of individuals needed to accurately estimate within or between populations *M*_e_ is currently unknown. It is also unknown if a discrepancy in number of genotyped individuals in two breeds affects the estimates of *M*_e_ between populations.

The main objective of our study was to investigate number of individuals needed to accurately estimate *M*_e_ within and between populations, and the size of difference in number of individuals in two breeds that allows for accurate estimation of between population *M*_e_. For this purpose, we simulated two populations that were separated by 100 generations. We evaluated how fast *M*_e_ changes across generations after separation and we also investigated if the absence of pedigree, a frequent occurrence in small breeds, affects the value of estimated *M*_e_. Finally, we studied the effect of marker density on the estimates of within and between population *M*_e_.

## 2 MATERIAL AND METHODS

### 2.1 Population structure

Two populations were simulated to reflect current domestic cattle breeds, specifically in terms of size of population, selection history and LD structure. These populations were related through common ancestry, originating from a historical population. The historical population consisted of 8,000 individuals in the base population. In the next 300 generations, population size gradually decreased (by ~25 individuals in each generation) to 400 individuals, and remained of such size for the following 20 generations, that is until generation 320. The bottle neck was used to achieve LD. From generation 320 until generation 340, the population size gradually increased to 5,000 individuals. Number of males in generation 340 was 50; number of females was 4,950. The genome consisted of 30 chromosomes, each of 100 cM. A total of 720,000 SNP markers were distributed equally and randomly over the chromosomes so that each chromosome contained 24,000 markers, similar to the high density Bovine BeadChip. As most traits of economic importance are quantitative traits, and to ensure a sufficient number of segregating QTL in the final data, the number of simulated QTLs was high, that is 9,000, which were equally distributed over the chromosomes, so that each chromosome contained 300 QTLs. QTLs were randomly distributed across the genome and their effects followed a gamma distribution with a shape parameter of 0.4. SNPs and QTLs had equal allele frequencies in the base generation of the historical population. The mutation rate of QTLs and markers was set to 2.5 × 10^–5^. All markers and QTLs were segregating in the last historical population.

The last generation of the historical population (i.e. generation 340) was randomly divided into two equally sized populations (A and B), so‐called founder populations, of each 2,500 individuals. In the next generation, the size of both populations was increased to 5,000, and in each population, 30 breeding males and 2,500 breeding females were available to produce 5,000 individuals for the next generation. Total number of individuals was kept constant for the following 100 generations. Number of offspring per female was set to 2, with 1:1 sex ratio. Throughout these 100 generations, both populations underwent selection based on EBVs, estimated from a best linear unbiased prediction method via an animal model, using phenotypic records and pedigree data. In each generation, 12 males and 500 females were replaced with individuals with the highest EBVs (a replacement ratio of 0.4 for the males and of 0.2 for the females). Thus, overlapping generations were present in the data. Selected males and females were randomly mated to each other, keeping the number of matings per male on average ~83.

Simulations were performed using QMSim software (Sargolzaei & Schenkel, 2009) and consisted of 10 replicates. Appendix S1 contains the QMSim parameter file, and Appendix S2 contains the seed file used for simulation.

### 2.2 Estimating *M*_e_

Different approaches can be applied to estimate within population *M*_e_, relying on either *N*_e_ or on the variation in genomic relationships between the individuals (Goddard, 2009; Goddard et al., 2011; Hayes, Visscher, & Goddard^2009^). In this study, we used the latter (see Discussion). The within population *M*_e_ was estimated using the following equation (Goddard et al., 2011; Wientjes et al., 2013) :

(1) $$M_{e}\;=\;\frac{1}{\text{Var}\;(G_{\mathrm{ij}}‐A_{\mathrm{ij}})}$$

where *G_ij_* is the genomic and *A_ij_* is the pedigree relationship between individual *i* and *j*, and the variance is taken over all pairs *ij* in the population. In analogy to this equation, *M*_e_ between populations can be estimated as follows (Wientjes et al., 2013):

(2) $$M_{e}\;=\;\frac{1}{\text{Var}\;{\text{(G}}_{{\text{pop1}}_{i}\;{\text{pop2}}_{j}}‐A_{\text{pop}1_{i}\;\text{pop}2_{j}})}$$

where $G_{{\text{pop1}}_{i}\;{\text{pop2}}_{j}}$ is the genomic relationship between individual *i* from population 1 and individual *j* from population 2, and $A_{\text{pop}1_{i}\;\text{pop}2_{j}}$ is the corresponding pedigree relationship, with the variance taken across all pairs of individuals from population 1 and 2. Conceptually, two populations can be considered as one reference population and *M*_e_ is estimated as the effective number of chromosome segments that are segregating in the combined population (Wientjes et al., 2016). The genomic relationship between unrelated individuals is expected to be 0 (Goddard et al., 2011).

The *M*_e_ was estimated with calc_grm software (Calus & Vandenplas, 2016), using an exponential function to adjust **G**‐**A** values to be on average 0 across the range of pedigree relationship values (Wientjes et al., 2016). The matrix **G** was calculated using following equation $G\;=\;\left[\begin{array}{cc} G_{11} & G_{12}\\ G_{21} & G_{22} \end{array}\right]\;=\;\left[\begin{array}{cc} \frac{Z_{1}Z_{1}^{\prime }}{\sum 2p_{1k}\;\left(1‐p_{1k}\right)} & \frac{Z_{1}Z_{2}^{\prime }}{\sqrt{\sum 2p_{1k}\;\left(1‐p_{1k}\right)}\sqrt{\sum 2p_{2k}\;\left(1‐p_{2k}\right)}}\\ \frac{Z_{2}Z_{1}^{\prime }}{\sqrt{\sum 2p_{1k}\;\left(1‐p_{1k}\right)}\sqrt{\sum 2p_{2k}\;\left(1‐p_{2k}\right)}} & \frac{Z_{2}Z_{2}^{\prime }}{\sum 2p_{2k}\;\left(1‐p_{2k}\right)} \end{array}\right]$ where **G**_11_ is a matrix with genomic relationships in population 1, **G**_22_ is a matrix with genomic relationships in population 2, while **G**_12_ and **G**_21_ are matrices with genomic relationships between population 1 and 2 (Wientjes et al., 2016). **Z**_1_ (**Z**_2_) matrix contains genotypes for all individuals from population 1 (population 2) at all loci, centred by subtracting twice the allele frequency per locus, and *p*_1_ *_k_* (*p*_2_ *_k_*) is the allele frequency of marker *k* in the population 1 (population 2). $Z_{1}Z_{2}^{\prime }$ and $Z_{2}Z_{1}^{\prime }$ are matrices of genetic covariance between the genetic values of two populations, divided by the SDs of the genotypes in each population $\sqrt{\sum 2p_{1k}\;\left(1‐p_{1k}\right)}$ and $\sqrt{\sum 2p_{2k}\;\left(1‐p_{2k}\right)}$.

### 2.3 Scenarios

To get insight into the effect of number of genotyped individuals used on the accuracy of estimated within population *M*_e_, we tested five different sample sizes of 10, 50, 100, 500 and 1,000 individuals, respectively. *M*_e_ was also estimated for the whole population of 5,000 individuals using 720k SNPs, which was considered closest to the true within *M*_e_ value, and was used for comparison with all other estimates. To test the effect of discrepancy in sample sizes from two populations on the accuracy of between *M*_e_, each sample size from each population was tested against each sample size from another population, resulting in 25 combinations in total. Similarly as for within *M*_e_, between *M*_e_ was also estimated using all 5,000 individuals from both breeds and 720k SNPs, and this estimate was used for comparison with all other estimates. All sampling of individuals was performed 50 times within each replicate, and the mean and standard deviation of 50 estimates of within and between *M*_e_ within a replicate were computed. Results are presented as averages of those means and standard deviations, across the 10 replicates. The estimates of *M*_e_ using all 5,000 individuals are presented as average values across the 10 replicates. The described estimation of *M*_e_ was done at generation 10, 50 and 100, in order to infer changes of *M*_e_ across generations. The pedigree consisted of 20,000 individuals that traced each population back four generations.

Detected levels of LD may be affected by marker density, such as SNPs compared to genome‐wide sequence data (Erbe et al., 2013; Qanbari et al., 2014), which subsequently can effect estimates of *M*_e_. In the default scenario, we simulated 720k SNPs at the last historical population, to reflect high marker density used in dairy cattle. To study the influence of different marker densities, we reduced the number of markers to subsets of 360, 180, 90, 45, 22.5 and 11.25k, which was achieved by selecting every 2*^x^*‐th marker, where *x* ranged from 1 to 6.

Calculation of *M*_e_ with Equations 1 and 2 requires pedigree to estimate additive genetic relationships between pairs of individuals in the same or between different populations. When this information is missing, *M*_e_ may be underestimated, especially for within population *M*_e_. For between *M*_e_, absence of pedigree may be less of an issue, since depending on the distance between the breeds, no or only a small number of individuals may have recent ancestry with individuals from another breed. We investigated the effect of pedigree absence on the estimation of *M*_e_ at generation 10, 50 and 100 after the split of the two breeds.

## 3 RESULTS

### 3.1 Summary statistics

In the last historical population, all 720k SNPs and 9,000 QTLs were still segregating. At generation 100 after the split, across 10 replicates, on average 7,256 (*SD* ± 684) SNPs in the population 1 and 7,299 (*SD* ± 791) SNPs in the population 2 were not segregating. An effective population size of 119 was estimated based on the sex ratio, $N_{e}\;=\;\frac{4\times N_{m}\times N_{f}}{N_{m}+N_{f}}$ (Wright, 1990), where *N*_m_ is the number of breeding males and *N*_f_ the number of breeding females. This value of *N*_e_ is close to those found in previous empirical cattle studies, where *N*_e_ was approximately 100 (Hall, 2016; Leroy et al., 2013). The squared correlation between pairs of SNPs (*r* ^2^) (Hill & Robertson, 1968) had on average (±*SD*) a value of 0.22 ± 0.23 at pairwise distances of 20–30 kb and 0.18 ± 0.20 at 60–70 kb for generation 100 for both populations, similar to observed LD patterns in real cattle populations (Qanbari et al., 2009, 2014). Allele frequencies of the SNPs followed the U‐shape distribution.

### 3.2 Within population *M*_e_

**FIGURE 1:**
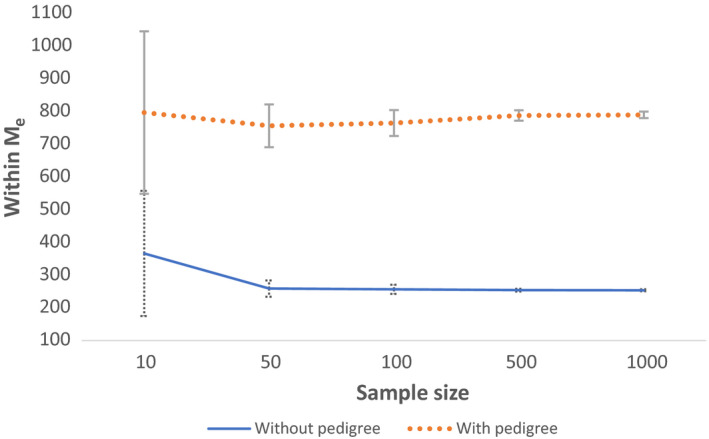
Within population *M*_e_ across different sample sizes in generation 100, estimated with 720k SNPs [Colour figure can be viewed at wileyonlinelibrary.com]

Table 1 presents estimates of within *M*_e_ in the whole population, at different SNP densities and at generations 10, 50 and 100. Since population 1 and 2 had similar estimates, because they had the same population history, one value was presented in the table, which was calculated as average within *M*_e_ of the two populations, with the average standard deviation. Using the whole population of 5,000 individuals, 720k SNPs, and no pedigree, estimated within *M*_e_ value across 10 replicates was 254 (*SD* ± 7) in generation 100. Regardless of the SNP density, similar values of *M*_e_ were obtained when the number of sampled individuals was 50 or higher; however, when the number of individuals was 10, within *M*_e_ was overestimated, with average estimated value of 361 (Figure 1 and Table 2). In addition, with 10 individuals, across replicates the average standard deviation of the estimated *M*_e_ was large, 189. Within *M*_e_ was overestimated and showed high variation when number of individuals was 10, regardless whether *M*_e_ was estimated at generation 10, 50 (Appendix S3) or 100 generations after splitting the populations (Table 2). These results indicate that at least 50 individuals are needed for accurate estimates of within *M*_e_ and that decreasing SNP density had a very small effect on the estimated *M*_e_.

**TABLE 1:** Estimates of within population *M*_e_ with and without pedigree, across generations and SNP densities using all 5,000 individuals

SNP density	Without pedigree			With pedigree		
	Gen 10	Gen 50	Gen 100	Gen 10	Gen 50	Gen 100
720k	298 (5)^a^	268 (7)	254 (7)	1,387 (69)	887 (26)	776 (44)
360k	298 (5)	268 (7)	254 (7)	1,385 (69)	885 (26)	775 (44)
180k	298 (5)	268 (7)	254 (7)	1,380 (68)	884 (26)	773 (44)
90k	298 (5)	267 (7)	254 (7)	1,370 (68)	881 (26)	770 (43)
45k	297 (5)	267 (7)	253 (7)	1,353 (66)	873 (27)	765 (43)
22.5k	295 (5)	267 (7)	252 (7)	1,315 (63)	860 (27)	754 (42)
11.25k	291 (5)	263 (6)	249 (7)	1,249 (57)	831 (26)	733 (36)

^a^ Estimates are presented as an average from 10 simulation replicates rounded to the closest number, and subsequently averaged over population 1 and 2, with standard deviation of a replicate between the brackets, also averaged over two populations.

**TABLE 2:** Estimates of within population *M*_e_ at generation 100, across different sample sizes and SNP densities

SNP density	Without pedigree (generation 100)					With pedigree (generation 100)				
	Sample size									
	10	50	100	500	1,000	10	50	100	500	1,000
720k	364 (192)	260 (26)	258 (15)	254 (5)	254 (3)	791 (253)	750 (63)	761 (39)	779 (16)	781 (10)
360k	363 (192)	260 (26)	257 (15)	254 (5)	254 (3)	791 (253)	749 (63)	760 (39)	778 (15)	780 (10)
180k	363 (191)	260 (26)	257 (15)	254 (5)	254 (3)	789 (252)	748 (63)	759 (39)	777 (15)	779 (10)
90k	362 (190)	259 (25)	257 (15)	254 (5)	253 (3)	786 (250)	745 (63)	756 (39)	774 (15)	775 (10)
45k	362 (190)	259 (25)	256 (14)	253 (5)	253 (3)	781 (247)	740 (62)	750 (39)	768 (15)	770 (10)
22.5k	359 (186)	258 (25)	255 (14)	252 (5)	252 (3)	770 (241)	731 (61)	741 (38)	758 (15)	759 (10)
11.25k	354 (181)	256 (25)	252 (14)	250 (4)	249 (3)	750 (234)	711 (58)	720 (36)	736 (14)	738 (10)

Note

Estimates are averages over 10 simulation replicates, averaged over population 1 and 2. Within each replicate sampling and *M*_e_ estimation has been repeated 50 times, and average *M*_e_ and standard deviation of a replicate have been calculated.

Standard deviations of a replicate are given in brackets as an average over 10 simulation replicates, averaged over population 1 and 2.

Appendix S3 contains estimates of within population *M*_e_ for generation 10 and 50.

**FIGURE 2:**
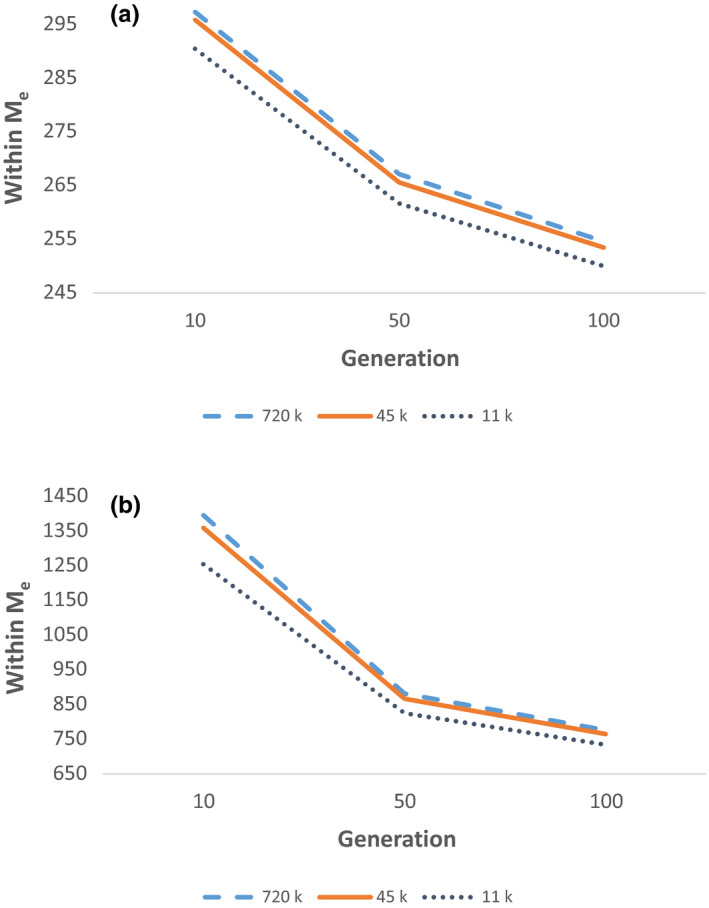
Within population *M*_e_ across generations on the example of 720, 45 and 11.25k SNPs, estimated with (a) and without (b) pedigree and using the whole population [Colour figure can be viewed at wileyonlinelibrary.com]

With pedigree, estimated within *M*_e_ was ~3*x* higher in both populations, 776 (*SD* ± 44) on average, at generation 100 when 720k SNPs were used. When pedigree was included, estimates of within *M*_e_ were slightly more affected by SNP density (Table 1). With the smallest sample size, within *M*_e_ on average had similar value as other sample sizes; however, variation around the mean remained high (Figure 1, Table 2, Appendix S3). Across generations, within *M*_e_ values showed a decreasing trend in all scenarios (Figure 2).

### 3.3 Between population *M*_e_

**FIGURE 3:**
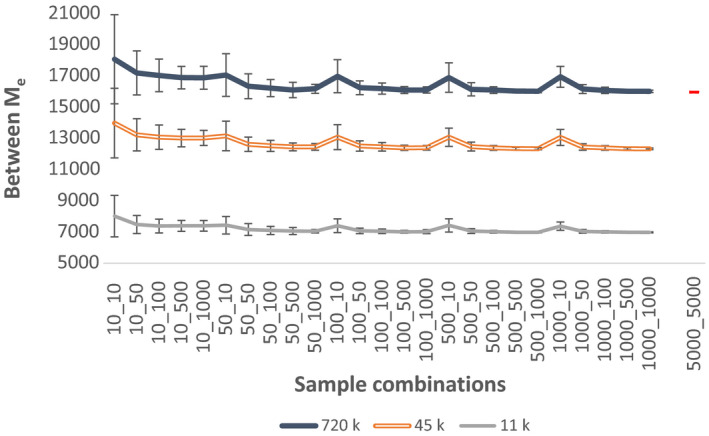
Estimates of between population *M*_e_ (with standard deviations of a replicate) across different sample combinations in generation 100, using 720k. For a comparison, figure also includes estimate of between population *M*_e_ using all individuals (5000_5000) and 720k SNPs

The estimated *M*_e_ between the two populations using all individuals, 720k SNPs and no pedigree, was 16,036 (*SD* ± 529) at generation 100 (Table 3). Unlike for estimation of within *M*_e_, where different SNP densities had small effect, between *M*_e_ was highly influenced by number of available SNPs (Table 3). For example, at generation 100, using 45k SNPs between *M*_e_ was underestimated by 23%, and the lowest SNP density of ~11k SNPs, often used to genotype cows, underestimated between *M*_e_ by more than 46% (Figure 3, Appendix S4). Regardless of SNP density, when the number of sampled individuals was 50 or more in both populations, estimates of between *M*_e_ were close to that of the whole population. On the other hand, whenever one population had only 10 individuals, between *M*_e_ was on average overestimated with a large standard deviation (Figure 3, Appendix S4). These results suggest that at least 50 individuals from both populations are needed for accurate estimation of between *M*_e_.

**TABLE 3:** Estimates of between population *M*_e_, across generations and SNP densities using all 5,000 individuals

SNP density	Without pedigree		
	Gen 10	Gen 50	Gen 100
720k	7,117 (383)	11,874 (265)	16,036 (529)
360k	7,054 (376)	11,704 (252)	15,755 (492)
180k	6,926 (363)	11,367 (242)	15,134 (449)
90k	6,687 (338)	10,741 (199)	14,096 (386)
45k	6,258 (296)	9,682 (159)	12,351 (339)
22.5k	5,542 (228)	8,065 (109)	9,852 (246)
11.25k	4,505 (150)	6,044 (71)	6,988 (167)

Note

Estimates are presented as an average of estimates from 10 simulation replicates rounded to the closest number, with standard deviation of a replicate between the brackets.

From generation 10–100, between *M*_e_ increased by ~9,000 when 720k SNPs were used. Increase of between *M*_e_ is expected as populations diverge more in time, especially when there is no exchange of individuals, which was the case in our simulation. Since pedigree used had no shared ancestors in either 10 or 100 generations beyond the historical population, they effectively had pedigree based relationships of ~0, and between *M*_e_ estimates were the same as those without pedigree (results not showed).

## 4 DISCUSSION

In this study, we conducted a simulation that mimics current domestic cattle populations in order to investigate how estimated effective number of chromosome segments (*M*_e_), within and between populations, is affected by number of genotyped individuals, SNP density and pedigree availability. Our results show that a small sample of genotyped individuals is expected to lead to overestimation of *M*_e_ and therefore may not accurately represent population structure. Based on our findings, at least 50 genotyped individuals are needed for accurate estimation of both within and between population *M*_e_. While estimates of within population *M*_e_ were hardly affected by SNP density, between population *M*_e_ values were highly dependent on the number of available SNPs, with higher SNP densities being able to detect more independent chromosome segments. When pedigree was used, estimates of within population *M*_e_ were approximately three to four times higher than estimates with genotypes only; however, between *M*_e_ estimates remained the same. Although the two populations used here had a similar population history, in term of implications of our results it may equally well represent situations where the reference population of a local breed is complemented with animals from another local or mainstream breed. This is because the effective population size of the simulated populations of 118 (calculated based on the numbers of breeding males and females) is close to estimates for local and mainstream breeds.

### 4.1 Within population *M*_e_

Estimated within *M*_e_ using all individuals and no pedigree had a value of ~254 in both populations at generation 100. In previous studies on cattle populations, within *M*_e_ values varied significantly depending on the breed and the method used to estimate within *M*_e_ (Brard & Ricard, 2015). When formulas based on *N*_e_ were used, within *M*_e_ ranged between 800 and 8,000, based on the results from 76 studies (Brard & Ricard, 2015). Back‐solving *M*_e_ from deterministic formulas for genomic prediction accuracy, after equating those to empirical cross‐validation accuracies for milk yield and somatic cell score, yielded within *M*_e_ of ~1,000–2,000 for a Holstein Friesian population, and *M*_e_ values of 150–400 for Brown Swiss (Erbe et al., 2013). As *M*_e_ is linked to effective population size, it is expected that breeds with lower genetic diversity have smaller *M*_e_ values. In a recent study that analysed five numerically small Dutch Red cattle breeds, within *M*_e_ ranged between 100 and 300, corresponding to values in our simulation (Marjanovic et al., 2018). From generation 10–100, within *M*_e_ in our study decreased by ~50, which is expected since artificial selection reduces genetic variation and increases relatedness among individuals. Hence, empirical estimates of within *M*_e_ are expected to strongly depend on the selection history of the population.

When within *M*_e_ was estimated using pedigree, the values increased approximately fourfold at generation 10 and threefold at generation 100 in both populations. Estimated *M*_e_ of similar magnitude (~1,390 at generation 10) has been reported for a Holstein Friesian population, where *M*_e_ was computed using the same approach as in our study (Wientjes et al., 2016). Considering the computation using $M_{e}=\frac{1}{\text{Var}\;(G_{\mathrm{ij}}‐A_{\mathrm{ij}})}$, it is worthwhile noting that all variance in the genomic relationships is likely also present in the pedigree relationships, since *E* (**G**|**A**) = **A** (Goddard et al., 2011), meaning that Var (*A_ij_*) may be a lower limit of *E* (Cov (*G_ij_*; *A_ij_*)). Assuming $E\;\left(\text{Cov}\;\left(G_{\mathrm{ij}};A_{\mathrm{ij}}\right)\right)\;\approx \;\text{Var}\;(A_{\mathrm{ij}})$ for simplicity, we get: $\text{Var}\;\left(G_{\mathrm{ij}}\;‐\;A_{\mathrm{ij}}\right)\;=\;\text{Var}\;\left(G_{\mathrm{ij}}\right)\;‐\;2\;\text{Cov}\;\left(G_{\mathrm{ij}};A_{\mathrm{ij}}\right)\;+\;\text{Var}\;\left(A_{\mathrm{ij}}\right)\;\approx \;\text{Var}\;\left(G_{\mathrm{ij}}\right)\;‐\;\text{Var}\;\left(A_{\mathrm{ij}}\right),$ and $M_{e}\;\approx \;\frac{1}{\text{Var}\;\left(G_{\mathrm{ij}}\right)\;‐\;\text{Var}\;\left(A_{\mathrm{ij}}\right)}$. Within livestock populations relatively high relationships, such as those between full‐ and half‐sibs, parent–offspring, and parent–grand offspring, are abundant. The presence of such relationships will considerably add to the variance across all relationships in the population. The above reformulated equation for *M*_e_ clearly shows that the subtraction of the pedigree from the genomic relationships will considerably reduce the variance of the denominator, and thus increase the estimated *M*_e_.

In numerically small breeds, pedigrees may be incomplete or not available, which could result in underestimation of *M*_e_ and therefore overestimation of genomic prediction accuracy. In such cases where the aim is to predict the accuracy of within breed genomic prediction, it would be advisable to derive the pedigree from genotypic information, and use this to build the pedigree relationship matrix. Although such approach may result in incomplete pedigree if not all relationships are reconstructed. With incomplete pedigree, some pedigree relationships will incorrectly be considered zero, and therefore not appropriately corrected in G‐A, leading to increase in var(G‐A) and decrease in *M*_e_. The majority of small breeds, however, may require a multi‐breed reference population, which requires also the *M*_e_ values between breeds. Those are, however, not influenced by pedigree information unless recent introgression occurred, and in general can be safely computed while ignoring pedigree information.

We tested the effect of five different sample sizes on the estimates of within *M*_e_. When the number of genotyped individuals was more than 50, the estimates varied only slightly across 50 replicates, and average *M*_e_ corresponded to that from the whole population, both for scenarios with and without pedigree. However, when the sample size was 10, average *M*_e_ was substantially overestimated when pedigree was not used. A possible explanation is that with 10 animals, the relative contribution of high pedigree relationships to the term $\text{Var}\;(G_{\mathrm{ij}}\;‐\;A_{\mathrm{ij}})$ is greater than when a larger number of animals is selected, which inflates the *M*_e_ but gets corrected with the pedigree. Nevertheless, even when using the pedigree relationships, there was a large standard deviation of the *M*_e_ across iterations, suggesting that a single estimate based on 10 animals could still deviate considerably from the true value.

The within *M*_e_ value can be computed using different formulas. In our study, the within *M*_e_ was based on the variance of genomic relationships, and in some scenarios, the additive genetic relationships were used as well (Equation 1). This approach has two important benefits. Firstly, it can be extended to two breeds, allowing for computation of between *M*_e_, necessary for across‐breed prediction, which is not possible with other formulas. Other frequently used approaches rely on effective population size (*N*_e_) and size of the genome (*L*), for example $M_{e}\;=\;\frac{2N_{e}L}{\ln\;(4N_{e}L)}$ (Goddard, 2009) and *M*_e_ = 2*N*_e_ *L* (Hayes, Visscher, & Goddard^2009^) and eigen value decomposition of the genomic relationship matrix (Misztal, 2016; Pocrnic et al., 2016). The estimates from different formulas can vary considerably, consequently affecting predicted accuracy of genomic selection (Brard & Ricard, 2015). In addition, equations based on *N*_e_ introduce another variation, as *N*_e_ can be estimated in several different ways (Leroy et al., 2013; Wang et al., 2016). Secondly, computing *M*_e_ based on the variance of relationships enables to consider specific characteristics of a population, such as population structure, as disclosed by observed genotypes of the population. In a recent study by van den Berg et al. (2019), authors have found that prediction accuracy using within *M*_e_ from genomic relationship matrix resulted in overestimation of the accuracy. It should be noted, however, that *M*_e_ is not the only parameter affecting the accuracy of GP (Goddard, 2009; Wientjes, et al., 2015). Nevertheless, in the study by van den Berg et al. (2019), the true within *M*_e_ may have been underestimated due to close relationships among some animals in the reference population, which could also be expected in numerically small breeds. However, using breed‐specific allele frequencies, as done in our study, reduced overestimation for between *M*_e_.

### 4.2 Between population *M*_e_

At generation 100, between population *M*_e_ had a value of 16,036 (529) when all individuals and 720k SNPs were used. This value is ~63 times larger than *M*_e_ within population computed without pedigree, and ~21 times larger than within *M*_e_ estimated with pedigree. Larger between population *M*_e_ compared to within *M*_e_ is expected, since LD structure, upon which *M*_e_ is dependent, is at least partly different between the two populations, as generally observed between different breeds (De Roos et al., 2008; Wientjes, Calus, Goddard, & Hayes 2015; Wientjes, et al., 2015). Indeed, between *M*_e_ in a study on Groningen White Headed, Holstein Friesian, and Meuse‐Rhine‐Yssel (MRY) breed, was 10× higher than within *M*_e_, and ranged between 18,000 and 24,000 (Wientjes, et al., 2015). The between *M*_e_ value in our study increased by ~9,000 from generation 10–100, indicating that closely related breeds, that is those that have split recently, are expected to have smaller between *M*_e_ . Our recent study showed that *M*_e_ between MRY and Deep Red breed, which was derived from MRY, was ~3,600 but ~17,000 between these two breeds and distantly related Groningen White Headed (Marjanovic et al., 2018).

Single‐nucleotide polymorphisms densities used to compute between population *M*_e_ substantially affected its value, with higher number of SNPs giving higher between *M*_e_ value. This finding is related to the number of independent segments, which is much larger between breeds, than within the breed; hence, many more markers are needed to capture all independent segments.

### 4.3 Implications

One of the challenges of numerically small breeds is that in terms of performance, they may be lagging behind compared to mainstream breeds. In that respect, their survival can significantly be aided by using genomic selection to speed up genetic gain in those breeds, as an alternative to increasing revenues for instance by focusing on specific niche markets. Whether or not implementation of genomic selection for small breeds is cost‐effective, depends not only on the achieved additional genetic improvement, but also on the costs of the implementation. It has been suggested that genotype costs can be shared across multiple applications, including use in conservation programs to manage genetic diversity and control inbreeding (Fernández et al., 2016), and parentage and pedigree verification (Berry et al., 2016). Also, based on continuously dropping costs of genotyping, it has been envisaged that entire cattle populations, or at least large proportions thereof, may be routinely genotyped in the near future (Boichard et al., 2015). Aiming to overcome the limited additional genetic improvement due to the reference population size being restricted by limitations to investments or numbers of available animals within a small breed, in recent years a lot of research has been dedicated to the use of a multi‐breed reference population as an attractive approach to increase the accuracy of genomic prediction for numerically small populations (Hayes, Bowman, Chamberlain, Verbyla, & Goddard 2009; Hozé et al., 2014; Lund et al., 2016). In general, reliabilities of across‐breed predictions tend to be lower than within–breed genomic prediction, due to differences in LD structure, allele frequencies and independent chromosome segments between the breeds (De Roos et al., 2009; Wientjes, Calus, Goddard, & Hayes 2015). Close family relationships between the breeds are often missing, which further affects the reliabilities. High SNP density gives more accurate representation of consistency of LD phase across populations, which at short distances are expected to be conserved across populations (De Roos et al., 2008), possibly resulting in an increased accuracy. Our study showed that accurate computation of between *M*_e_ does require a SNP density higher than the common 50k. Genotyping individuals with high density SNP chips is more expensive compared to commonly used 50k SNP chip. Alternatively, if possible, individuals could be genotyped with lower SNP density and imputed to higher density, albeit the impact of using imputed genotypes on the estimated *M*_e_ is currently unknown. Nevertheless, high density genotyping will likely become more affordable in the coming years. Based on our results, no more than 50 individuals are required to be genotyped per population, to enable assessing the potential benefit of genomic selection for this population, which should help keeping the costs down.

## 5 CONCLUSIONS

In conclusion, our results showed that for accurate estimation of within and between population *M*_e_, 50 or more animals should be genotyped per population. Pedigree information was not relevant for between *M*_e_ in our simulation, which is expected to be also true for real populations, unless recent introgression occurred. Estimates of within *M*_e_ were highly affected by whether pedigree was used or not. For numerically small breeds, pedigree may often be absent, in which case a pedigree relationship matrix could be built using a pedigree derived from genotypic information. For within *M*_e_, even the smallest SNP densities resulted in accurate representation of family relationships in the population; however, for between *M*_e_, many more markers are needed to capture all independent segments. Presented findings can be used as guidelines for studies investigating possibilities for genomic predication in numerically small populations.

## CONFLICT OF INTEREST

The authors declare that they have no competing interests.

## AUTHOR CONTRIBUTIONS

MPLC and JM designed the study. JM performed the statistical analysis and drafted the manuscript. MPLC contributed to the interpretation of results and the writing of the manuscript. Both authors read and approved the final manuscript.

## Supporting information

Appendix S1

Appendix S2

Appendix S3

Appendix S4

## Data Availability

All information supporting the results is included in the text, figures and tables of this article. The data sets can be generated using Appendix S1 and S2.
