# Supplementary material for: Factors affecting accuracy of estimated effective number of chromosome segments for numerically small breeds
Source: J Anim Breed Genet. 2020 Oct 10;138(2):151–60. doi: 10.1111/jbg.12512 (PMC7891385; doi:10.1111/jbg.12512)
Supplement: Supplementary file 3 — Appendix S3 [file JBG-138-151-s003.docx]

**Supplementary file 3**

| Table S1 Estimates of within population $M_{e}$ at generation 10, across different sample sizes and SNP densities | | | | | | | | | | | |
| --- | --- | --- | --- | --- | --- | --- | --- | --- | --- | --- | --- |
| **SNP density** | **Without pedigree (generation 10)** | | | | |  | **With pedigree (generation 10)** | | | | |
|  | **Sample size** | | | | | | | | | | |
|  | **10** | **50** | **100** | **500** | **1000** |  | **10** | **50** | **100** | **500** | **1000** |
| 720k | 488 (335) | 309 (34) | 301 (18) | 299 (6) | 299 (4) |  | 1307 (499) | 1288 (171) | 1331 (122) | 1371 (59) | 1390 (58) |
| 360k | 487 (334) | 309 (33) | 301 (18) | 299 (6) | 299 (4) |  | 1306 (499) | 1286 (170) | 1329 (122) | 1370 (58) | 1389 (57) |
| 180k | 485 (334) | 308 (33) | 301 (18) | 299 (6) | 298 (4) |  | 1303 (495) | 1282 (170) | 1325 (121) | 1367 (58) | 1384 (56) |
| 90k | 485 (334) | 308 (32) | 300 (18) | 298 (6) | 298 (4) |  | 1291 (489) | 1273 (167) | 1315 (119) | 1358 (57) | 1374 (56) |
| 45k | 482 (329) | 307 (32) | 300 (17) | 298 (6) | 298 (4) |  | 1279 (483) | 1260 (166) | 1299 (117) | 1342 (56) | 1357 (55) |
| 22.5k | 476 (314) | 305 (32) | 298 (18) | 296 (6) | 296 (4) |  | 1246 (462) | 1227 (160) | 1264 (110) | 1305 (54) | 1318 (53) |
| 11.25k | 464 (298) | 301 (31) | 294 (17) | 292 (6) | 292 (4) |  | 1195 (435) | 1171 (150) | 1204 (101) | 1243 (52) | 1252 (49) |
| Estimates are averages over 10 simulation replicates, averaged over population 1 and 2. Within each replicate sampling and $M_{e}$ estimation has been repeated 50 times, and average $M_{e}$ and standard deviation of a replicate have been calculated.  Standard deviations of a replicate are given in brackets as an average over 10 simulation replicates, averaged over population 1 and 2. | | | | | | | | | | | |

| Table S2 Estimates of within population $M_{e}$ at generation 50, across different sample sizes and SNP densities | | | | | | | | | | | |
| --- | --- | --- | --- | --- | --- | --- | --- | --- | --- | --- | --- |
| **SNP density** | **Without pedigree (generation 50)** | | | | |  | **With pedigree (generation 50)** | | | | |
|  | **Sample size** | | | | | | | | | | |
|  | **10** | **50** | **100** | **500** | **1000** |  | **10** | **50** | **100** | **500** | **1000** |
| 720k | 395 (224) | 275 (28) | 272 (15) | 268 (5) | 268 (3) |  | 897 (270) | 847 (74) | 863 (46) | 882 (19) | 885 (12) |
| 360k | 394 (223) | 275 (28) | 271 (15) | 268 (5) | 268 (3) |  | 895 (270) | 846 (74) | 862 (46) | 881 (19) | 884 (12) |
| 180k | 393 (222) | 275 (28) | 271 (15) | 268 (5) | 268 (3) |  | 893 (268) | 843 (74) | 859( 46) | 878 (18) | 881 (12) |
| 90k | 393 (222) | 274 (28) | 271 (15) | 267 (5) | 267 (3) |  | 890 (268) | 837 (73) | 854 (45) | 872 (18) | 875 (12) |
| 45k | 391 (222) | 274 (28) | 270 (15) | 266 (5) | 266 (3) |  | 880 (267) | 827 (72) | 843 (44) | 862 (18) | 864 (12) |
| 22.5k | 387 (218) | 271 (28) | 268 (15) | 264 (4) | 264 (3) |  | 862 (269) | 809 (70) | 824 (43) | 841 (18) | 843 (12) |
| 11.25k | 383 (211) | 270 (27) | 266 (15) | 263 (4) | 263 (3) |  | 850 (256) | 796 (66) | 811 (41) | 827 (17) | 829 (12) |
| Estimates are averages over 10 simulation replicates, averaged over population 1 and 2. Within each replicate sampling and $M_{e}$ estimation has been repeated 50 times, and average $M_{e}$ and standard deviation of a replicate have been calculated.  Standard deviations of a replicate are given in brackets as an average over 10 simulation replicates, averaged over population 1 and 2. | | | | | | | | | | | |
