# Supplementary material for: Factors affecting accuracy of estimated effective number of chromosome segments for numerically small breeds
Source: J Anim Breed Genet. 2020 Oct 10;138(2):151–60. doi: 10.1111/jbg.12512 (PMC7891385; doi:10.1111/jbg.12512)
Supplement: Supplementary file 4 — Appendix S4 [file JBG-138-151-s004.docx]

| Table S3 Estimates of between population $M_{e}$, across sample combinations and SNP densities in generation 10 | | | | | | | | | | |
| --- | --- | --- | --- | --- | --- | --- | --- | --- | --- | --- |
| **Sample combinations** | | |  | **SNP density** | | | | | | |
| **Pop 1** | **Pop 2** | |  | **720k** | **360k** | **180k** | **90k** | **45k** | **22.5k** | **11.25k** |
| 10 | | 10 |  | 8094 (1548) | 8030 (1558) | 7882 (1516) | 7583 (1418) | 7088 (1329) | 6296 (1137) | 5099 (853) |
| 10 | | 50 |  | 7679 (684) | 7613 (684) | 7480 (670) | 7216 (645) | 6731 (583) | 5978 (496) | 4863 (379) |
| 10 | | 100 |  | 7677 (574) | 7604 (565) | 7464 (543) | 7202 (515) | 6719 (475) | 5944 (413) | 4816 (312) |
| 10 | | 500 |  | 7623 (427) | 7562 (403) | 7424 (391) | 7172 (373) | 6704 (341) | 5925 (290) | 4806 (234) |
| 10 | | 1000 |  | 7571 (482) | 7535 (406) | 7395 (394) | 7136 (374) | 6671 (337) | 5903 (291) | 4799 (238) |
| 50 | | 10 |  | 7726 (697) | 7666 (675) | 7529 (656) | 7277 (638) | 6801 (570) | 6010 (485) | 4881 (383) |
| 50 | | 50 |  | 7335 (329) | 7270 (324) | 7137 (315) | 6889 (297) | 6436 (275) | 5691 (235) | 4627 (187) |
| 50 | | 100 |  | 7249 (395) | 7219 (264) | 7091 (254) | 6848 (244) | 6408 (224) | 5669 (203) | 4603 (142) |
| 50 | | 500 |  | 7205 (394) | 7198 (184) | 7064 (179) | 6820 (169) | 6375 (157) | 5648 (146) | 4598 (177) |
| 50 | | 1000 |  | 7230 (293) | 7197 (176) | 7064 (172) | 6821 (165) | 6379 (148) | 5651 (142) | 4588 (106) |
| 100 | | 10 |  | 7649 (592) | 7598 (546) | 7461 (535) | 7195 (500) | 6719 (451) | 5938 (369) | 4816 (283) |
| 100 | | 50 |  | 7260 (373) | 7228 (230) | 7100 (221) | 6851 (212) | 6408 (196) | 5678 (178) | 4612 (139) |
| 100 | | 100 |  | 7256 (255) | 7206 (199) | 7073 (194) | 6830 (182) | 6383 (167) | 5653 (151) | 4600 (127) |
| 100 | | 500 |  | 7230 (158) | 7172 (121) | 7043 (119) | 6802 (117) | 6354 (105) | 5628 (101) | 4577 (95) |
| 100 | | 1000 |  | 7282 (121) | 7219 (119) | 7084 (115) | 6835 (108) | 6393 (100) | 5653 (84) | 4577 (64) |
| 500 | | 10 |  | 7565 (456) | 7503 (440) | 7364 (430) | 7111 (407) | 6649 (366) | 5883 (312) | 4774 (241) |
| 500 | | 50 |  | 7200 (195) | 7136 (192) | 7008 (186) | 6764 (177) | 6329 (161) | 5604 (133) | 4554 (105) |
| 500 | | 100 |  | 7005 (322) | 6962 (281) | 6898 (137) | 6667 (134) | 6264 (154) | 5580 (188) | 4535 (176) |
| 500 | | 500 |  | 7059 (75) | 6999 (74) | 6873 (72) | 6636 (68) | 6217 (61) | 5514 (51) | 4488 (37) |
| 500 | | 1000 |  | 7056 (63) | 6995 (61) | 6868 (59) | 6632 (56) | 6214 (52) | 5510 (43) | 4485 (32) |
| 1000 | | 10 |  | 7495 (430) | 7441 (393) | 7303 (378) | 7052 (359) | 6608 (325) | 5864 (274) | 4761 (209) |
| 1000 | | 50 |  | 7156 (183) | 7094 (181) | 6965 (178) | 6727 (170) | 6297 (155) | 5582 (133) | 4540 (94) |
| 1000 | | 100 |  | 7232 (136) | 7167 (135) | 7036 (132) | 6793 (125) | 6352 (113) | 5623 (91) | 4565 (65) |
| 1000 | | 500 |  | 7195 (69) | 7131 (68) | 7000 (66) | 6758 (62) | 6319 (56) | 5591 (45) | 4539 (32) |
| 1000 | | 1000 |  | 7180 (90) | 7122 (50) | 6992 (50) | 6750 (49) | 6313 (45) | 5589 (53) | 4535 (25) |
| Estimates are averages over 10 simulation replicates. Within each replicate sampling and $M_{e}$ estimation has been repeated 50 times, and average $M_{e}$ and standard deviation of a replicate have been calculated.  Standard deviations of a replicate are given in brackets as an average over 10 simulation replicates. | | | | | | | | | | |

**Supplementary file 4**

| Table S4 Estimates of between population $M_{e}$, across sample combinations and SNP densities in generation 50 | | | | | | | | | |
| --- | --- | --- | --- | --- | --- | --- | --- | --- | --- |
| **Sample combinations** | |  | **SNP density** | | | | | | |
| **Pop 1** | **Pop 2** |  | **720k** | **360k** | **180k** | **90k** | **45k** | **22.5k** | **11.25k** |
| 10 | 10 |  | 13360 (2262) | 13156 (2215) | 12749 (2161) | 12041 (2039) | 10881 (1784) | 9077 (1512) | 6840 (1162) |
| 10 | 50 |  | 12711 (1065) | 12543 (1008) | 12186 (971) | 11514 (924) | 10365 (805) | 8649 (684) | 6459 (504) |
| 10 | 100 |  | 12644 (830) | 12495 (743) | 12135 (723) | 11445 (660) | 10320 (606) | 8607 (532) | 6427 (404) |
| 10 | 500 |  | 12557 (625) | 12399 (529) | 12039 (504) | 11377 (469) | 10259 (448) | 8539 (394) | 6406 (300) |
| 10 | 1000 |  | 12535 (703) | 12408 (486) | 12047 (475) | 11389 (454) | 10276 (416) | 8552 (322) | 6409 (270) |
| 50 | 10 |  | 12724 (991) | 12536 (963) | 12174 (942) | 11497 (900) | 10358 (800) | 8617 (690) | 6446 (483) |
| 50 | 50 |  | 12155 (441) | 11978 (436) | 11633 (422) | 10977 (407) | 9890 (358) | 8247 (287) | 6182 (215) |
| 50 | 100 |  | 12079 (345) | 11907 (339) | 11565 (328) | 10923 (307) | 9849 (273) | 8199 (226) | 6143 (164) |
| 50 | 500 |  | 12009 (215) | 11837 (212) | 11496 (202) | 10863 (193) | 9793 (175) | 8157 (148) | 6110 (106) |
| 50 | 1000 |  | 11990 (321) | 11840 (190) | 11501 (186) | 10865 (174) | 9791 (156) | 8165 (172) | 6118 (138) |
| 100 | 10 |  | 12609 (836) | 12449 (741) | 12103 (717) | 11432 (674) | 10301 (619) | 8560 (543) | 6430 (399) |
| 100 | 50 |  | 12036 (379) | 11876 (314) | 11533 (302) | 10894 (288) | 9823 (256) | 8181 (213) | 6139 (175) |
| 100 | 100 |  | 11996 (255) | 11825 (249) | 11485 (242) | 10856 (225) | 9786 (205) | 8148 (166) | 6110 (145) |
| 100 | 500 |  | 11941 (157) | 11770 (153) | 11429 (148) | 10800 (138) | 9740 (130) | 8113 (103) | 6079 (76) |
| 100 | 1000 |  | 11935 (137) | 11765 (136) | 11427 (132) | 10798 (123) | 9735 (113) | 8114 (91) | 6080 (66) |
| 500 | 10 |  | 12573 (501) | 12396 (494) | 12038 (476) | 11368 (460) | 10250 (411) | 8546 (330) | 6394 (251) |
| 500 | 50 |  | 12022 (240) | 11849 (236) | 11505 (225) | 10870 (213) | 9799 (183) | 8164 (149) | 6118 (105) |
| 500 | 100 |  | 11945 (167) | 11774 (165) | 11431 (158) | 10801 (147) | 9738 (129) | 8114 (108) | 6079 (77) |
| 500 | 500 |  | 11874 (239) | 11728 (84) | 11390 (82) | 10761 (75) | 9699 (67) | 8078 (54) | 6056 (40) |
| 500 | 1000 |  | 11890 (72) | 11720 (70) | 11382 (67) | 10755 (63) | 9696 (55) | 8076 (45) | 6052 (33) |
| 1000 | 10 |  | 12597 (492) | 12417 (485) | 12058 (469) | 11383 (453) | 10253 (411) | 8538 (337) | 6404 (248) |
| 1000 | 50 |  | 12008 (210) | 11840 (207) | 11497 (200) | 10866 (191) | 9797 (168) | 8162 (130) | 6117 (98) |
| 1000 | 100 |  | 11913 (287) | 11753 (209) | 11426 (141) | 10799 (138) | 9739 (143) | 8107 (93) | 6089 (122) |
| 1000 | 500 |  | 11843 (332) | 11721 (69) | 11385 (71) | 10756 (71) | 9701 (91) | 8098 (166) | 6067 (138) |
| 1000 | 1000 |  | 11886 (59) | 11716 (58) | 11379 (56) | 10753 (58) | 9693 (58) | 8074 (57) | 6049 (27) |
| Estimates are averages over 10 simulation replicates. Within each replicate sampling and $M_{e}$ estimation has been repeated 50 times, and average $M_{e}$and standard deviation of a replicate have been calculated.  Standard deviations of a replicate are given in brackets as an average over 10 simulation replicates. | | | | | | | | | |

| Table S5 Estimates of between population $M_{e}$, across sample combinations and SNP densities in generation 100 | | | | | | | | | |
| --- | --- | --- | --- | --- | --- | --- | --- | --- | --- |
| **Sample combinations** | |  | **SNP density** | | | | | | |
| **Pop 1** | **Pop 2** |  | **720k** | **360k** | **180k** | **90k** | **45k** | **22.5k** | **11.25k** |
| 10 | 10 |  | 18068 (2856) | 17762 (2771) | 17097 (2679) | 15942 (2507) | 13978 (2232) | 11209 (1823) | 8023 (1328) |
| 10 | 50 |  | 17194 (1411) | 16889 (1365) | 16245 (1300) | 15116 (1167) | 13231 (1030) | 10551 (809) | 7485 (578) |
| 10 | 100 |  | 17034 (1048) | 16738 (1019) | 16082 (990) | 14964 (916) | 13076 (782) | 10395 (612) | 7387 (442) |
| 10 | 500 |  | 16891 (720) | 16600 (698) | 15951 (691) | 14860 (636) | 13019 (560) | 10377 (442) | 7398 (353) |
| 10 | 1000 |  | 16883 (734) | 16608 (629) | 15963 (621) | 14886 (571) | 13031 (486) | 10419 (442) | 7399 (343) |
| 50 | 10 |  | 17055 (1367) | 16812 (1232) | 16147 (1164) | 15029 (1096) | 13156 (947) | 10498 (820) | 7439 (562) |
| 50 | 50 |  | 16338 (796) | 16095 (614) | 15466 (585) | 14429 (548) | 12625 (459) | 10060 (392) | 7165 (381) |
| 50 | 100 |  | 16220 (548) | 15963 (433) | 15338 (427) | 14290 (409) | 12520 (354) | 9995 (336) | 7104 (264) |
| 50 | 500 |  | 16097 (494) | 15874 (294) | 15252 (282) | 14217 (281) | 12456 (250) | 9944 (270) | 7068 (223) |
| 50 | 1000 |  | 16173 (289) | 15896 (283) | 15272 (273) | 14228 (250) | 12455 (210) | 9940 (189) | 7052 (107) |
| 100 | 10 |  | 16981 (1058) | 16681 (1030) | 16036 (1001) | 14940 (931) | 13080 (802) | 10394 (600) | 7405 (439) |
| 100 | 50 |  | 16241 (441) | 15964 (435) | 15338 (412) | 14281 (394) | 12512 (332) | 9974 (261) | 7077 (179) |
| 100 | 100 |  | 16189 (358) | 15909 (347) | 15278 (328) | 14236 (309) | 12459 (274) | 9934 (208) | 7050 (144) |
| 100 | 500 |  | 16097 (224) | 15817 (218) | 15194 (209) | 14158 (195) | 12398 (165) | 9885 (125) | 7018 (91) |
| 100 | 1000 |  | 16103 (201) | 15827 (196) | 15203 (188) | 14163 (174) | 12399 (150) | 9883 (118) | 7025 (125) |
| 500 | 10 |  | 16895 (943) | 16654 (761) | 15997 (722) | 14917 (678) | 13074 (585) | 10433 (460) | 7425 (424) |
| 500 | 50 |  | 16143 (425) | 15886 (305) | 15262 (290) | 14220 (269) | 12476 (286) | 9950 (232) | 7064 (152) |
| 500 | 100 |  | 16092 (214) | 15813 (210) | 15188 (195) | 14150 (178) | 12390 (150) | 9886 (120) | 7021 (89) |
| 500 | 500 |  | 16026 (113) | 15749 (111) | 15128 (106) | 14095 (97) | 12346 (84) | 9842 (66) | 6991 (45) |
| 500 | 1000 |  | 16021 (99) | 15743 (97) | 15122 (93) | 14090 (84) | 12337 (71) | 9838 (58) | 6987 (39) |
| 1000 | 10 |  | 16942 (670) | 16654 (658) | 15987 (640) | 14897 (594) | 13060 (513) | 10398 (380) | 7380 (265) |
| 1000 | 50 |  | 16160 (285) | 15881 (277) | 15254 (263) | 14216 (246) | 12451 (207) | 9923 (168) | 7046 (117) |
| 1000 | 100 |  | 16076 (202) | 15797 (199) | 15173 (190) | 14141 (171) | 12389 (147) | 9874 (114) | 7012 (80) |
| 1000 | 500 |  | 16023 (97) | 15746 (94) | 15125 (89) | 14092 (84) | 12340 (71) | 9838 (56) | 6987 (41) |
| 1000 | 1000 |  | 16013 (75) | 15735 (73) | 15114 (69) | 14085 (64) | 12332 (55) | 9829 (44) | 6981 (31) |
| Estimates are averages over 10 simulation replicates. Within each replicate sampling and $M_{e}$estimation has been repeated 50 times, and average $M_{e}$and standard deviation of a replicate have been calculated.  Standard deviations of a replicate are given in brackets as an average over 10 simulation replicates. | | | | | | | | | |
